# Sleep Problems and Mental Health in Middle‐Aged and Older Autistic and Non‐Autistic Adults

**DOI:** 10.1002/gps.70259

**Published:** 2026-10-07

**Authors:** Sophie Roper, Andreea Barbinta, Alice M. G. Quinton, Rebecca A. Charlton, Francesca Happé, Gavin R. Stewart

**Affiliations:** ^1^ Social, Genetic and Developmental Psychiatry Centre Institute of Psychiatry, Psychology & Neuroscience King's College London London UK; ^2^ Department of Psychology Goldsmiths University of London London UK

**Keywords:** autism, mental health, midlife, old age, sleep

## Abstract

**Background:**

Sleep is a vital biological function, and impaired sleep can lead to a range of problems, including poor health and wellbeing. Poor sleep and higher rates of sleep‐related problems and difficulties are often reported by autistic people of all ages, however, these experiences and problems have seldom been studied in diagnosed autistic populations in midlife and old age.

**Methods:**

This study used data from the first wave of the AgeWellAutism cohort, from a sample of 265 autistic and 167 age‐ and gender‐matched non‐autistic adults aged 40–93 years (mean = ∼60 years; ∼50% female). Participants completed widely used and standardised measures of sleep quality and mental health symptoms.

**Results:**

The autistic group reported significantly greater sleep problems than the non‐autistic group, with poorer overall sleep across all domains (all *p* < 0.001), with autistic women reporting greatest difficulties. The largest differences between groups were observed in sleep disturbances and daytime dysfunction (*χ* ^2^ = 101.8 and 95.1, respectively, both *p* < 0.001). These differences remained significant after controlling for age and symptoms of anxiety, depression and PTSD. Across both groups, poorer sleep was associated with older age and higher levels of anxiety, depression and PTSD symptoms, with a stronger association between sleep problems and anxiety symptoms in the autistic group.

**Conclusions:**

Consistent with prior autism trait‐based research in the general population, middle‐aged and older autistic adults report more extensive sleep problems and worse sleep quality than non‐autistic adults. Sleep problems were associated with older age and poorer mental health. Though the study is cross‐sectional, the findings highlight the need to explore causal links through intervention. Addressing anxiety, and mental health problems more broadly, may improve sleep quality in ageing autistic populations.

## Key Points

- Middle‐aged and older autistic adults report significantly more sleep problems than non‐autistic peers, including poor sleep quality, longer sleep onset, and increased daytime fatigue, even after accounting for age and mental health problems.
- Poor sleep in both autistic and non‐autistic individuals was associated with older age and poorer mental health, specifically symptoms of depression, anxiety, and PTSD.
- Sleep problems had a stronger relationship with anxiety symptoms in the autistic group versus the non‐autistic group, suggesting anxiety may play an important role in sleep quality among ageing autistic individuals.
- The findings emphasise the need for targeted interventions to address anxiety and other mental health concerns to potentially improve sleep—and related outcomes in middle‐aged and older autistic adults.

## 1 Introduction

Autism is a neurodevelopmental condition, estimated to affect around 1% of the global population, characterised by differences in social communication and rigid‐repetitive behaviours [1, 2]. Given increasing recognition of autism across the lifespan and population ageing, a substantial proportion of autistic people are now middle‐aged and older adults, yet this group has often been overlooked [3]. Specifically, only 0.4% of indexed autism research between 1980 and 2020 focused on this older age group [4]. A growing evidence‐base reveals that autistic people of all ages experience on average greater health burdens, poorer mental health, poorer normative life outcomes [4, 5, 6, 7, 8, 9, 10, 11, 12], more periods of crisis [13, 14, 15, 16], and lower quality of life [17, 18, 19], than their non‐autistic peers.

Autistic children and adults have also often been found to experience problems with sleep [20, 21, 22, 23, 24]. Sleep is a vital biological function [25], with disrupted sleep predicting early mortality in the general population [26]. Experiences of sleep change with age, with older adults often experiencing sleep problems, such as shorter sleep duration and more fragmented sleep, contributing to increased daytime fatigue, impaired cognitive function, mood disturbances and lower quality of life [27]. Research indicates that sleep problems are particularly common among autistic individuals [20, 28]. A meta‐analysis by Morgan et al. (2020) [22] reviewed 14 empirical studies of autistic adults (mean age 21–40 years, total *n* = 194), finding more significant subjectively and objectively measured sleep problems compared to non‐autistic peers. Subjective complaints (measured by self‐report) included lower sleep efficiency (i.e., more time awake relative to sleep time in bed), longer sleep onset latency (i.e., taking longer to fall asleep), and higher prevalence of sleep problems. Objective problems (measured by actigraphy or polysomnography) included lower sleep efficiency, greater sleep fragmentation, longer sleep onset latency, and shorter total sleep time. Qualitative studies also document persistent sleep problems and their association with poorer health and wellbeing in autistic adults [21, 24]. However studies have largely focused on autistic young adults, and neglected midlife and old age.

Although sleep problems are widely reported in autistic populations, it is important to distinguish between *sleep problems* and *sleep disorders*. Sleep problems typically refer to symptoms such as prolonged sleep onset latency, night waking, reduced sleep duration, and poor sleep efficiency, while sleep disorders are clinically defined conditions classified in diagnostic systems such as the DSM‐5 and ICSD‐3, including insomnia disorder and circadian rhythm sleep–wake disorders [1, 29]. While many studies report on sleep problems and subjective symptoms like prolonged sleep onset latency or frequent night wakings, there is also evidence for higher rates of clinically defined sleep disorders in autistic populations. Lai et al. (2019) [30] reported significantly higher rates of sleep disorders in autistic adults, though specific diagnoses were often not differentiated. Where specific disorders are identified, insomnia remains the most common; however, research by Baker (2017) [31] and Hare et al. (2006) [32] suggests that autistic individuals may also be more prone to specific circadian sleep‐wake disorders. These clinical trends are particularly evident in healthcare record analyses. Croen et al. (2015) [6], analysing private healthcare records from 1507 individuals aged 19–79, found higher rates of insomnia and dyssomnia in autistic adults compared to matched non‐autistic peers (17.5% vs. 9.6% OR = 1.9). Similarly, Bishop‐Fitzpatrick et al. (2017) [5] reported that 85% of autistic adults aged 40+ had a dyssomnia diagnosis in Medicaid records. Dyssomnia as an umbrella term, covering a range of conditions including insomnia and hypersomnia, rather than as a single specific diagnosis. Taken together, these cross‐sectional findings highlight a significant clinical burden in middle‐aged and older autistic adults, though longitudinal studies are required to track how these diagnostic patterns shift across the lifespan.

Beyond clinical diagnoses, the impact of sleep on the daily lives of autistic adults is profound. Sleep disruption is not merely a co‐occurring symptom but a critical predictor of lower quality of life, affecting daily functioning and general life satisfaction [33]. Furthermore, sleep may act as a key mechanism that explains poor mental health in autistic populations; for instance, research indicates that sleep quality significantly mediates the relationship between autistic traits and depressive symptoms [34]. This suggests that the high rates of depression seen in autistic populations may be partially driven by chronic sleep disruption. These relationships are further complicated by social and environmental factors, with sleep quality being closely intertwined with levels of social support and experiences of loneliness [35], which have been found to be more common in autistic populations [10, 36]. This cycle between sleep and mental health is most evident when examining symptoms of anxiety and depression, which are reported at significantly higher rates in autistic adults than in the general population [37, 38, 39]. However, to better understand sleep impairment in this group, it is essential to consider the role of trauma. Autistic individuals may be at a heightened risk for Post‐Traumatic Stress Disorder (PTSD) due to increased exposure to social and environmental stressors throughout the lifespan [40, 41, 42]. PTSD is uniquely relevant here because its core symptoms, specifically physiological hyperarousal and nightmares, directly exacerbate the sleep‐onset difficulties often attributed to generalised anxiety [43, 44]. By examining PTSD symptoms alongside depression and anxiety, this study provides a more nuanced investigation into how various forms of psychological distress may impact sleep problems in ageing autistic populations.

Although research specifically targeting older autistic populations remains limited, recent lifespan studies and trait‐based research in the general population have begun to illuminate these age effects. Jovevska et al. (2020) [45], examining individuals aged 15–80, found that autistic participants, particularly those in midlife (aged 40–59), reported significantly poorer sleep quality and longer sleep onset latency than non‐autistic peers. This pattern is mirrored in older adults with high autistic traits; Stewart et al. (2020) [23] identified that despite having similar total sleep durations to their low‐trait peers, high‐trait individuals aged 50–81 experienced markedly lower sleep satisfaction, greater difficulty falling asleep, and a higher prevalence of severe sleep problems (21.4% vs. 10.3%). Across these studies, specific demographic and clinical factors emerge as key predictors of sleep dysfunction. Notably, Jovevska et al. (2020) [45] identified being female as the strongest predictor of poor sleep, while also noting the significant role of medication use. Similarly, Charlton et al. (2023) [45] found that female gender (sex assigned at birth), physical health problems, and anxiety symptoms predicted poor sleep in a large autistic sample (*N* = 730). While Charlton et al. found that age had little impact on global sleep quality, older age specifically correlated with greater sleep disturbance, a finding that highlights the increased difficulties faced by autistic individuals as they age. Crucially, these sleep disturbances appear to have a disproportionately negative impact on mental health. Stewart et al. (2020) [23] demonstrated a significant interaction where individuals with both high autistic traits and severe sleep problems exhibited the highest levels of depression and anxiety among all groups. This suggests that sleep may not only be a symptom of psychological distress but a factor that exacerbates it. While these papers suggest higher rates of sleep problems in midlife and older age, the fact that mean participant ages in diagnostic studies often remain below 50 highlights the need for research specifically tailored to the unique clinical and biological experiences of autistic populations in later life [45, 46]. These gaps have important implications for gerontology and geriatric psychiatric practice. Sleep disturbance, depression, anxiety, and trauma‐related symptoms are associated with poorer physical health, cognitive functioning, and quality of life in later adulthood [26, 27, 47, 48, 49, 50]. Improved understanding of sleep and psychological distress in older autistic adults may therefore support more accurate assessment, tailored intervention, and age‐sensitive mental healthcare provision for this growing population.

The current study is an expansion of the previously mentioned Stewart et al. (2020) [23] trait‐based study, examining sleep problems and mental health in middle‐aged and older autistic adults versus age‐ and gender‐matched non‐autistic adults. We predict that autistic adults will report (i) higher sleep problems, including more people above the suggested clinical cut‐off for sleep problems; (ii) higher symptom levels of depression, anxiety, and PTSD; and (iii) stronger associations between sleep problems and depression, anxiety and PTSD symptom severity compared with non‐autistic adults. Further, (iv) older autistic adults (age 65+) will report higher rates of sleep problems and poorer self‐reported symptoms of depression, anxiety and PTSD in comparison to midlife (age 40–64) autistic adults. Finally, (v) women will report higher rates of sleep problems and poorer self‐reported symptoms of depression, anxiety, and PTSD than men, with this effect being stronger in the autistic group than non‐autistic group.

## 2 Methods

### 2.1 Study Design

This pre‐registered study (https://osf.io/973z8/) uses cross‐sectional data from the first wave of the ‘AgeWellAutism’ study, conducted in Spring 2019. The AgeWellAutism study is an online survey investigating ageing on the autism spectrum (outlined in Stewart et al. (2024)) [10]. In brief, the AgeWellAutism study was steered by 12 middle‐aged and older autistic adults prior to launch, with recruitment conducted via social media platforms, research databases, Autistica's Research Network, and advertisements in community centres and older adult residential communities. Participants were entered into a draw for one of twenty £20 Amazon UK gift vouchers. Inclusion criteria were being 40 years of age or above, having access to an internet‐enable device, and being able to read and type in English. The study had no specific exclusion criteria. Full ethical approval was granted by the PNM Research Ethics Subcommittee at King's College London (HR‐18/19–10941).

### 2.2 Participants

In total, 502 completed surveys were recorded, with 70 responses excluded due to suspected spam (i.e., very short completion times and/or irregular answers to open‐text boxes). The final sample had 432 participants aged 40–93 years. Participants who disclosed that they either had an autism diagnosis (*n* = 254) or self‐identified as autistic (*n* = 11) formed an autistic group (*n* = 265); both subgroups had comparable Ritvo Autism and Asperger Diagnostic Scale (RAADS‐14) [51] autistic trait scores (*t*(1,263) = 1.54, *p =* 0.124), with mean scores in both subgroups exceeding the cut‐off for clinically significant autistic traits. Timing of autism diagnoses/self‐identification ranged from as recently as the year of survey completion to 43 years earlier (mean years since diagnosis = 10.3 years), with 17 (6.4%) diagnosed in childhood (i.e., prior to 18 years of age). The remaining participants formed the non‐autistic group (*n* = 167). Inclusion of both clinically diagnosed and self‐identified autistic participants was specified a priori within the study protocol to improve inclusivity and capture potentially underdiagnosed older autistic adults [3, 52]. All participants in the autistic group (diagnosed and self‐identified) scored above established screening thresholds on the RAADS‐14, supporting the validity of group classification. The autistic and non‐autistic groups were matched on age (autistic mean age = 60.5 years; non‐autistic mean age = 60.6 years) and gender ratio (autistic group men = 46.8%; non‐autistic group men = 50.3%). Both groups were comparable in education level and in country of residence, with around 98% living in the UK. The autistic group self‐reported lower employment rates and a higher likelihood of living with non‐marital family members. Information on socio‐economic status and race/ethnicity data were not collected in the survey.

For age group analyses, age was dichotomised into midlife (aged 40–64 years; autistic *n* = 158, non‐autistic *n* = 106) and old age (aged 65+; autistic *n* = 107, non‐autistic *n* = 61) subgroups.

See Table 1 for demographic characteristics of the autistic and non‐autistic groups.

**TABLE 1:** Demographic characteristics of the autistic and non‐autistic groups.

		Autistic group (*n* = 265)		Non‐autistic group (*n* = 167)		Group difference	Effect size
Age (years)	*M* (SD)	60.59 (12.89)		60.53 (13.54)		F(1,430) = 0.01, *p =* 0.960	*d* = 0.01 [−0.19–0.19]
	[95% CI]	[59.03–62.15]		[58.46–62.60]			
	Min‐max	40–91		40–93			
Gender	men: women: nb/t	124:137: 4		84:83		*χ* ^2^ = 2.86, *p =* 0.239	*d* = 0.10 [−0.09–0.29]
	%	46.8%: 51.7%: 1.5%		50.3%: 49.7%			
Living situation	Spouse or partner	98 (37.0%)		79 (47.3%)		*χ* ^2^ = 4.52, *p =* 0.034^a^	*v* = 0.102
	Children	71 (26.8%)		39 (23.4%)		*χ* ^2^ = 0.64, *p =* 0.424	*v* = 0.038
	Sibling	34 (12.8%)		0		*χ* ^2^ = 23.26, *p <* 0.001***	*v* = 0.232
	Parent	22 (8.3%)		7 (4.2%)		*χ* ^2^ = 2.76, *p =* 0.096	*v* = 0.0.80
	Other family member	10 (3.8%)		0		*χ* ^2^ = 6.45, *p =* 0.011*	*v* = 0.122
	Roommate/friend	21 (7.9%)		12 (7.2%)		*χ* ^2^ = 0.08, *p =* 0.778	*v* = 0.014
	Supported housing	44 (16.6%)		20 (12.0%)		*χ* ^2^ = 1.74, *p =* 0.187	*v* = 0.063
	Alone independently	52 (19.6%)		58 (34.7%)		*χ* ^2^ = 12.32, *p <* 0.001***	*v* = 0.169
Education history	No formal qualifications	28	10.6%	4	2.4%	*χ* ^2^ = 1.05, *p =* 0.310	*v* = 0.209
	School to 16	62	23.40%	27	16.2%		
	School to 18	77	29.10%	66	39.5%		
	Undergraduate	59	22.3%	51	30.5%		
	Postgraduate	39	14.7%	19	11.4%		
Current employment status	Employed	75	(28.3%)	88	(52.7%)	*χ* ^2^ = 45.65, *p <* 0.001***	*v* = 0.325
	Retired	125	(47.2%)	74	(44.3%)		
	Unemployed	65	(24.5%)	5	(3.0%)		
Autism diagnosis	Diagnosed	254	(95.8%)	0	—	—	—
	Self‐identified	11	(4.2%)	0			
Years since autism diagnosis/identity	*M* (SD)	10.26	(8.22)	—		—	—
	Min‐Max	0–43					

Abbreviation: nb/t = non‐binary and trans.

^a^ Does not survive FDR correction.

FDR adjusted a‐value = *p < 0.029, **p < 0.01, ***p < 0.001.

### 2.3 Materials

#### 2.3.1 Demographic Characteristics

Participants provided detailed self‐reported demographic information, including age, gender, highest educational attainment, employment status, who they live with, and country of residence. Participants were also asked whether they had an autism diagnosis or if they self‐identified as autistic. If yes, they were then asked when they received their diagnosis or began to self‐identify.

#### 2.3.2 Sleep

Sleep problems were measured using the Pittsburgh Sleep Quality Index (PSQI) questionnaire [53], a 24‐item questionnaire assessing objective sleep behaviours and subjective difficulties over the past month. The first 18 PSQI items are self‐report, and can be used to calculate seven subscales (overall sleep quality, sleep latency, sleep duration, sleep efficiency, sleep disturbance, requiring sleep medication and daytime dysfunction), scored from 0 (no problems) to 3 (greatest dysfunction). One item asks if they have a bedpartner, and if so, five further PSQI items rated by the participant's bedpartner are included for clinical purposes but are not included in the scoring. These subscales are totalled to produce a global sleep problem score (range: 0–21), with a cut‐off score ≥ 6 indicating sleep disturbances. In the current sample, the internal consistency of the PSQI was very good in the autistic group (Cronbach's *α* = 0.82) and good in the non‐autistic group (Cronbach's *α* = 0.78). Previous research further indicates the PSQI is reliable in autistic adults, with reported internal consistency of *α* = 0.75 [46].

#### 2.3.3 Depression

Symptoms of depression were measured using the Patient Health Questionnaire (PHQ‐9) [54], a nine‐item questionnaire using a four‐point scale, assessing a range of problems over the past 2 weeks. Using the conventional cut‐off score of ≥ 10, the PHQ‐9 has a sensitivity of 88% and a specificity of 88% for major depressive disorder. The PHQ‐9 has been validated for use in autistic adults [55]. In the current sample, the internal consistency of the PHQ‐9 was very good in the autistic group (Cronbach's *α* = 0.88) and good in the non‐autistic group (Cronbach's *α* = 0.71).

#### 2.3.4 Anxiety

Symptoms of anxiety were measured using the Generalised Anxiety Disorder questionnaire (GAD‐7) [56]. The GAD‐7 is a seven‐item questionnaire using a four‐point scale assessing the severity of anxiety symptoms over the past 2 weeks. Using the conventional cut‐off score of ≥ 10, the GAD‐7 has a sensitivity of 89% and a specificity of 82% for generalised anxiety disorder. The GAD‐7 has been validated for use in autistic younger adult populations [57]. In the current sample, the internal consistency of the GAD‐7 was acceptable in both autistic (Cronbach's *α* = 0.78) and non‐autistic groups (Cronbach's *α* = 0.74).

#### 2.3.5 PTSD

Symptoms of PTSD were measured using the abbreviated Post Traumatic Stress Disorder Checklist‐Civilian (PCL‐6) [58]. The PCL‐6 is a six‐item questionnaire using a 5‐point scale assessing PTSD symptom frequency in the past month. Using the conventional cut‐off of ≥ 17, the PCL‐6 has a sensitivity of 80% and a specificity of 76% for PTSD. To the authors knowledge, the PCL‐6 has not yet been validated in autistic samples, however, the longer 20‐item PCL‐5 has been found to have excellent internal consistency in autistic populations (Cronbach's *α* = 0.92–0.94) [59]. In the current sample, the internal consistency of the PCL‐5 was excellent in the autistic group (Cronbach's *α* = 0.90) and good in the non‐autistic group (Cronbach's *α* = 0.79).

### 2.4 Data Analysis

All analyses were conducted using SPSS (v25.0; IBM Corp., 2017). As outlined above, sleep problems and poor mental health were measured through self‐reported symptom severity rather than formal clinical diagnosis. Group differences (autistic vs. non‐autistic) in demographics were assessed using *t*‐tests (continuous variables) and chi‐square (*χ* ^2^) tests (categorical variables). *χ* ^2^ tests examined differences in sleep problem subscales between autism groups (hypothesis 1), as well as gender (men vs. women; hypothesis 5) and age (midlife, age 40–64 vs. old age, age 65+; hypothesis 4), with adjusted residuals identifying specific differences. While dichotomizing age in this manner reduces statistical power, an a priori decision was made to include additional analysis with this stratification to produce outputs specific to older autistic people and to improve clinical interoperability for those working in older adult services. Additionally, this stratification avoids assuming a linear age‐outcome relationship. ANOVAs assessed group differences in total sleep quality index scores (hypothesis 1), as well as symptoms of depression, anxiety, and PTSD (hypothesis 2). ANCOVAs further adjusted sleep quality index scores for symptoms of depression, anxiety, PTSD, and age to assess whether between‐group differences in sleep persist after accounting for mental health symptom severity. Bivariate correlations (with Fisher's r‐to‐z transformations) examined associations between sleep quality index scores and mental health symptoms within each group (hypothesis 3). Gender and age group effects and interactions on sleep problem subscales and sleep quality index scores were explored using layered *χ* ^2^ tests and 2 × 2 ANOVA (hypothesis 4 and 5).

To reduce Type I error associated with multiple comparisons, False Discovery Rate (FDR) correction using the Benjamini–Hochberg procedure [60] was applied to all analyses conducted. The resulting FDR‐adjusted significance threshold for the primary analyses was *p* ≤ 0.029.

The online survey used prompted‐response items for all questionnaire measures; participants were unable to progress with unanswered items. Consequently, there was no missing data for the analysed variables among completed surveys, and no imputation procedures were required.

## 3 Results

### 3.1 Sleep Problems (PSQI Domain Scores)

The autistic group were significantly more likely to report difficulties across all PSQI domain scores compared to the non‐autistic group. These difficulties included poorer sleep quality (*χ* ^2^ = 71.1, *p <* 0.001), longer sleep latency (*χ* ^2^ = 24.2, *p <* 0.001), shorter sleep duration (*χ* ^2^ = 15.6, *p <* 0.001), lower sleep efficiency (*χ* ^2^ = 21.0, *p <* 0.001), more sleep disturbances (*χ* ^2^ = 101.8, *p <* 0.001), greater use of sleep medication (*χ* ^2^ = 27.5, *p <* 0.001), and more often experiencing daytime tiredness and dysfunction (*χ* ^2^ = 95.1, *p <* 0.001). Adjusted residual values indicate that the autistic group more frequently reported moderate‐to‐severe difficulties in these sleep domains than the comparison group. See Table 2.

**TABLE 2:** Frequencies and group differences of sleep problem domains of the autistic and non‐autistic groups.

		Sleep problems score					Group difference	Effect size
		No difficulty	Some difficulty	Moderate difficulty	Severe difficulty			
Sleep quality	Autistic	139 (52.7%)^a^	89 (33.7%)^a^	36 (13.6%)^a^	0	—	*χ* ^2^ = 71.1, *p <* 0.001***	*v* = 0.406
	Non‐Autistic	153 (91.6%)^a^	12 (7.2%)^a^	2 (1.2%)^a^	0	—		
Sleep latency	Autistic	14 (5.3%)	32 (12.1%)^a^	141 (53.4%)	77	(29.2%)	*χ* ^2^ = 24.2, *p <* 0.001***	*v* = 0.237
	Non‐Autistic	6 (3.6%)	52 (31.1%)^a^	75 (44.9%)	34	(20.4%)		
Sleep duration	Autistic	119 (45.1%)^a^	79 (29.9%)	53 (20.1%)^a^	13	(4.9%)^a^	*χ* ^2^ = 15.6, *p <* 0.001***	*v* = 0.190
	Non‐Autistic	97 (58.1%)	50 (29.9%)	20 (12.0%)^a^	0	—		
Sleep efficiency	Autistic	142 (53.8%)	63 (23.9%)^a^	59 (22.3%)^a^	0	—	*χ* ^2^ = 21.0, *p <* 0.001***	*v* = 0.221
	Non‐Autistic	92 (55.1%)	63 (37.7%)^a^	12 (7.2%)^a^	0	—		
Sleep disturbance	Autistic	4 (1.5%)^a^	121 (45.8%)^a^	125 (47.3%)^a^	14	(5.3%)^a^	*χ* ^2^ = 101.8, *p <* 0.001***	*v* = 0.485
	Non‐Autistic	28 (16.8%)^a^	124 (74.3%)^a^	15 (9.0%)^a^	0	—		
Medication use	Autistic	151 (57.2%)^a^	88 (33.3%)^a^	25 (9.5%)^a^	0	—	*χ* ^2^ = 27.5, *p <* 0.001***	*v* = 0.251
	Non‐Autistic	134 (80.2%)^a^	31 (18.6%)^a^	2 (1.2%)^a^	0	—		
Daytime dysfunction	Autistic	9 (3.4%)^a^	117 (44.4%)	118 (44.1%)^a^	20	(8.0%)^a^	*χ* ^2^ = 95.1, *p <* 0.001***	*v* = 0.471
	Non‐Autistic	54 (32.3%)^a^	86 (51.5%)	25 (15.0%)^a^	2	(1.2%)^a^		

^a^ Indicates significant adjusted residual value.

FDR adjusted a‐value = *p < 0.029, **p < 0.01, ***p < 0.001.

When considering gender differences, autistic women reported significantly greater difficulties in sleep duration (*χ* ^2^ = 13.39, *p =* 0.004), sleep disturbance (*χ* ^2^ = 11.39, *p =* 0.010) and daytime dysfunction (*χ* ^2^ = 13.38, *p =* 0.004) compared to autistic men. Adjusted residual values indicate that autistic women more frequently experienced moderate‐to‐severe difficulties in these sleep domains than autistic men. No other gender differences were found in the autistic group, and no gender differences were found in the non‐autistic group. See Supporting Information S1: Table S1.

### 3.2 Sleep Problems (PSQI Global Scores)

When viewing sleep problems as a combined composite score (i.e., PSQI global score), the autistic group reported significantly higher sleep quality index scores than the non‐autistic group (F(1,430) = 121.29, *p <* 0.001), indicating more cumulative sleep problems. Additionally, a significantly higher proportion of the autistic group were above cut‐off on the PSQI global score than the non‐autistic group (76.9% vs. 38.9%, respectively; *χ* ^2^ = 62.72, *p <* 0.001). See Table 3.

**TABLE 3:** Descriptive statistics and group differences of sleep problems and mental health problems of the autistic and non‐autistic groups.

		Autistic group (*n* = 265)	Non‐autistic group (*n* = 167)	Group difference	Effect size
Sleep quality index (max score = 21, cut‐off ≥ 6)	*M* (SD)	7.85 (2.95)	4.95 (2.12)	F(1,430) = 121.29, *p <* 0.001***	*d* = 1.12 [0.88–1.29]
	[95% CI]	[7.50–8.21]	[4.63–5.28]		
	Min‐max	1–16	1–14		
	% above cut‐off	203 (76.9%)	65 (38.9%)	*χ*2 = 62.72, *p <* 0.001***	*v* = 0.381
	*r* Depression	*r =* 0.386***	*r =* 0.259***	*z* = 1.43, *p =* 0.152	—
	*r* Anxiety	*r =* 0.459***	*r =* 0.186*	*z* = 3.27, *p <* 0.001***	
	*r* PTSD	*r =* 0.364***	*r =* 0.221**	*z* = 1.57, *p =* 0.116	
	*r* Age	*r =* 0.345***	*r =* 0.226**	*z* = 1.30, *p =* 0.193	
Depression symptoms (max score = 27, cut‐off ≥ 10)	*M* (SD)	9.82 (5.39)	2.99 (2.39)	F(1,430) = 237.35, *p <* 0.001	*d* = 1.52 [1.30–1.74]
	[95% CI]	[9.16–10.46]	[2.62–3.36]		
	Range	0–18	0–13		
	% above cut‐off	50 (18.9%)	11 (6.6%)	*χ* ^2^ = 12.74, *p <* 0.001***	*v* = 0.558
Anxiety symptoms (max score = 21, cut‐off ≥ 10)	*M* (SD)	6.36 (3.36)	2.49 (1.60)	F(1,430) = 192.98, *p <* 0.001	*d* = 1.37 [1.15–1.58]
	[95% CI]	[5.95–6.76]	[2.23–2.73]		
	Range	0–18	0–11		
	% above cut‐off	94 (35.5%)	13 (7.8%)	*χ* ^2^ = 42.14, *p <* 0.001***	*v* = 0.228
PTSD symptoms (max score = 24, cut‐off ≥ 17)	*M* (SD)	12.17 (6.32)	3.35 (3.65)	F(1,430) = 267.31, *p <* 0.001***	*d* = 1.61 [1.39–1.84]
	[95% CI]	[11.40–12.93]	[2.80–3.92]		
	Range	0–24	0–23		
	% above cut‐off	71 (26.8%)	2 (1.2%)	*χ* ^2^ = 47.79, *p <* 0.001***	*v* = 0.333

FDR adjusted a‐value = *p < 0.029, **p < 0.01, ***p < 0.001.

While a significant main effect of gender was initially found (women > men), this did not survive multiple comparison correction (F(1,423) = 4.26, *p =* 0.040). A significant interaction between autism group and gender (F(1,423) = 12.89, *p <* 0.001) indicated that within the autistic group, women reported significantly higher PSQI global scores than men, whereas no gender differences emerged in the non‐autistic group; see Supporting Information S1: Table S2. A similar pattern of results was found when accounting for age and current symptoms of depression, anxiety and PTSD. See Supporting Information S1: Tables S3 and S4.

### 3.3 Symptoms of Depression, Anxiety, and PTSD

The autistic group reported significantly higher symptoms of depression (F(1,430) = 237.35, *p <* 0.001), anxiety (F(1,430) = 192.98, *p <* 0.001), and PTSD (F(1,430) = 267.31, *p <* 0.001) than the non‐autistic group. Additionally, a significantly higher proportion of autistic versus non‐autistic individuals met the self‐report screener cut‐off thresholds for depression (18.9% vs. 6.6%; *χ* ^2^ = 12.74, *p <* 0.001), anxiety (35.5% vs. 7.8%; *χ* ^2^ = 42.14, *p <* 0.001) and PTSD symptoms (26.8% vs. 1.2%; *χ* ^2^ = 47.79, *p <* 0.001). See Table 3.

Few gender differences were found. A main effect of gender (women > men) was found in PTSD symptoms (F(1,423) = 42.90, *p <* 0.001). The gender difference in PTSD symptoms was significantly greater in the autistic group than in the non‐autistic group (F(1,423) = 9.97, *p =* 0.002), with the difference between women and men in PTSD scores being greater in the autistic group than non‐autistic group. No other main effects of gender or interactions were found in depression and anxiety scores; see Supporting Information S1: Table S2.

### 3.4 Associations Between Sleep Problems (PSQI Global Scores), Mental Health Symptoms, and Age

In the autistic group, PSQI global scores were significantly positively correlated with depression symptoms (*r =* 0.386, *p <* 0.001), anxiety symptoms (*r =* 0.459, *p <* 0.001), PTSD symptoms (*r =* 0.364, *p <* 0.001), and older age (*r =* 0.345, *p <* 0.001). Similarly, in the non‐autistic group, PSQI global scores were significantly positively associated with depression symptoms (*r =* 0.259, *p <* 0.001), anxiety symptoms (*r =* 0.186, *p <* 0.001), PTSD symptoms (*r =* 0.221, *p =* 0.004), and older age (*r =* 0.226, *p =* 0.003). The strength of association differed between autistic and non‐autistic groups only for PSQI global scores and anxiety symptoms (*z* = 3.27, *p <* 0.001). No group differences were found in the association strengths between PSQI global scores and depression symptoms, PTSD symptoms, or age.

### 3.5 Sleep Problems in Midlife and Old Age

When comparing midlife (age 40–64) and old age (age 65+) groups, differences in PSQI domain scores and PSQI global scores emerged within each group. Older autistic adults reported significantly higher rates of moderate and severe difficulties in the PSQI domain scores than middle‐aged autistic adults in sleep quality (*χ* ^2^ = 9.55, *p =* 0.008), sleep duration (*χ* ^2^ = 9.02, *p =* 0.029), sleep efficiency (*χ* ^2^ = 8.31, *p =* 0.016), sleep disturbances (*χ* ^2^ = 44.97, *p <* 0.001), requirement of sleep medicines (*χ* ^2^ = 8.05, *p =* 0.018), and daytime tiredness and dysfunction (*χ* ^2^ = 25.53, *p <* 0.001). No differences were found in sleep latency. Adjusted residual values indicate that the old age autistic sub‐group more often reported moderate‐to‐severe difficulties in these sleep domains than the midlife autistic sub‐group. Older non‐autistic adults also reported significantly more sleep problems than middle‐aged non‐autistic adults in sleep efficiency (*χ* ^2^ = 7.75, *p =* 0.021), sleep disturbances (*χ* ^2^ = 16.51, *p <* 0.001), and daytime tiredness and dysfunction (*χ* ^2^ = 9.79, *p =* 0.020). No differences were found in sleep quality, duration, or requirement of medication use. Adjusted residual values indicate that older non‐autistic adults more frequently experienced moderate‐to‐severe difficulties in these sleep domains than middle‐aged non‐autistic adults. See Supporting Information S1: Table S5.

When viewing sleep problems as a combined composite score (i.e., PSQI global score), a main effect of age group (old age > midlife; F(1,427) = 20.51, *p <* 0.001) was found. An interaction between autism group and age was also found (F(1,427) = 5.33, *p =* 0.021), with autism and old age having a compounded effect on sleep problems; see Supporting Information S1: Table S6. However, this interaction was not statistically significant when accounting for age and current symptoms of depression, anxiety and PTSD. See Supporting Information S1: Table S7.

## 4 Discussion

This study addresses a significant gap in the literature regarding sleep problems and associated mental health difficulties in a large sample of middle‐aged and older autistic adults. Our findings indicate that autistic adults aged 40–93 report significantly more severe and cumulative sleep problems than their age‐ and gender‐matched non‐autistic peers. These results provide a robust expansion of Stewart et al. (2020) [23], demonstrating that the relationship between heightened sleep problems and poor mental health, having been previously identified in high‐trait‐autistic populations, persists and remains clinically significant in a diagnosed autistic sample throughout the latter half of their lifespan. Notably, this difference remained even after controlling for age, anxiety, and depression, suggesting that poor sleep is not merely a byproduct of psychological distress, but may represent a core feature of the ageing autistic experience.

Consistent with previous research (e.g., Stewart et al. (2020); Jovevska et al. 2020) [23, 45], our results show that midlife and old age represent periods of significant sleep vulnerability for autistic individuals. While the correlation strength between age and sleep problems was similar in both the autistic and non‐autistic groups, the autistic group maintained a consistently higher burden of sleep problems. This pattern would align with a parallel effect, where both groups experience the normative age‐related changes in sleep, such as reduced melatonin secretion [61] and flattened circadian rhythms, but autistic individuals begin from a higher baseline of disruption [3].

In non‐autistic older adults, age‐related sleep disturbance is often attributed to primary ageing processes and increased physical health burden [62]. In contrast, these normative changes in autistic adults are likely compounded by autism‐specific factors, including sensory hypersensitivity that may worsen as coping resources decline with age [63, 64], and chronic physiological hyperarousal [65], reflecting prolonged exposure to social and environmental stressors that may maintain heightened physiological arousal. Further contributing factors in autistic adults may include greater physical and mental health burdens and increased medication use [66, 67, 68], all known to negatively impact sleep. Together, these biological, physiological, and social factors likely contribute to the heightened prevalence and severity of sleep problems in older autistic adults. Importantly, disrupted sleep is associated with increased mortality risk in the general population [26], highlighting the potential long‐term health implications of these findings. This highlights the potential clinical relevance of recognising sleep disturbance not as an isolated issue, but as a cumulative and interacting challenge across the lifespan in autistic individuals.

Further, poor sleep positively interacted with age, with older autistic adults reporting more difficulties than those in midlife. This likely reflects the combined effects of normative age‐related changes in sleep physiology, including reduced sleep efficiency and more fragmented sleep [69], alongside autism‐specific vulnerabilities. In later adulthood, additional age factors may further exacerbate sleep disruption, increased physical health burden and multimorbidity, as well as greater medication use, with commonly prescribed medications known to negatively impact sleep [70]. Reduced daily structure following retirement and weakened external time cue may also contribute to circadian rhythm dysregulation and greater sleep fragmentation in older age [71]. Together, these factors may contribute to a pattern whereby sleep problems become increasingly pronounced in later life for autistic adults.

Sleep problems were also moderated by gender, with autistic women reporting greater problems than autistic men. Such findings align with previous research (e.g., Jovevska et al. 2020) [45], which identified female sex as a significant predictor of poorer sleep outcomes, suggesting that gender‐related vulnerabilities may be amplified in autistic populations. Research indicates that autistic women are more likely to experience internalising difficulties, such as anxiety and depression, which are closely linked to sleep disruption and may contribute to sustained cognitive and physiological hyperarousal. In addition, autistic women are more likely to engage in social camouflaging, involving consciously masking or compensating for their autistic traits, than autistic men [72]. The cumulative impact of this increased masking has been associated with increased stress, exhaustion and poorer mental health, all of which may negatively affect sleep over time [72, 73]. Biological factors, including hormonal changes across the lifespan, including those occurring during menopause [74, 75, 76, 77] may further contribute to sleep disturbance for autistic women. Together, these interacting physiological, social, and biological factors likely contribute to the greater vulnerability to sleep problems observed in autistic women.

A further key finding was the strong association between sleep problems and mental health difficulties. This aligns with prior work in both the general population [78], and autistic/high autistic trait populations [23, 28, 45, 46]. While observed in both autistic and non‐autistic groups, the link with anxiety was significantly stronger for autistic participants. Studies consistently report strong associations between anxiety and sleep disruption in both autistic children and adults, indicating that this relationship may reflect a distinct, lifelong experience among autistic individuals [79, 80, 81, 82]. A potential explanation for the particularly strong association between anxiety and sleep problems in the autistic group can be understood through hyperarousal‐based models of sleep disturbance. Hyperarousal theories propose that insomnia and related sleep problems arise from heightened physiological and cognitive activation, including increased autonomic activity, persistent worry, and difficulty downregulating emotional responses. In autistic individuals, this state of hyperarousal may be especially pronounced due to heightened sensory sensitivity, intolerance of uncertainty, and a lifelong accumulation of stress related to navigating social and environmental demands [83]. Emerging evidence suggests that anxiety plays a central and persistent role in shaping mental health outcomes in midlife and older autistic adults, potentially acting as a maintaining factor rather than a co‐occurring condition [84]. Within this framework, anxiety may directly interfere with sleep initiation through cognitive processes such as rumination and anticipatory worry, while also contributing to nocturnal awakenings via physiological arousal [85]. In turn, poor sleep may further exacerbate anxiety by impairing emotional regulation and increasing reactivity to stress [86, 87], creating a self‐reinforcing cycle that may be particularly difficult to disrupt in autistic individuals. Despite this, sleep is rarely addressed systematically in clinical practice [88]. Explicitly assessing and targeting sleep problems in clinical practice may therefore represent a potentially valuable intervention pathway for reducing anxiety and improving anxiety outcomes in autistic adults [35].

When contextualising these findings, several strengths and limitations should be noted. A key strength was the involvement of autistic adults in shaping survey content and language use. The study also recruited a large, gender‐balanced sample of middle‐aged and older adults using multiple methods and included an age‐ and gender‐matched non‐autistic comparison group, enabling well‐powered analyses. Importantly, it contributes to an under‐researched area, as most autism research focuses on childhood or young adulthood, offering new insights into age‐related sleep problems.

However, it is important to note that, while the groups were matched on age and gender, the possibility remains that unmeasured factors may be driving associations observed. For example, social, health‐related (including medicines), and contextual differences between groups, such as socio‐economic status, ethnicity and access to healthcare, may have contributed to the observed differences in sleep outcomes. Additionally, factors such as employment status are difficult to account for in this sample due to the age range spanning from expected working age to expected retirement age. Further limitations include reliance on self‐report instruments (e.g., PSQI). While these capture perceived sleep quality and can predict functional difficulties [89], subjective reports may not align with objective measures such as actigraphy, with individuals often overestimating sleep latency and underestimating sleep time [90]. However, perceived sleep disturbance is itself clinically meaningful and closely linked to mental health outcomes, even when objective sleep appears relatively normal [91]. Future work should integrate both methods to clarify perceived versus actual sleep problems. The study also lacked detailed medication data (e.g., use of sleep aids or psychotropics), which could influence reported rates. Recruitment bias may further limit generalisability, as older research participants are typically healthier than the wider population [92]. Finally, the cross‐sectional design precludes conclusions about age‐related changes or causal links between sleep and mental health, as such the directionality or bidirectionality of findings in this study cannot be inferred [93]. Longitudinal studies combining validated self‐report tools, objective measures, medication data, and representative samples are needed to clarify these associations in ageing autistic populations.

In conclusion, this study replicates and expands on existing findings that autistic adults in midlife and old age experience significantly higher rates of sleep problems and mental health symptoms compared to non‐autistic adults. Sleep problems were not only more common but also more severe and cumulative in the autistic group, and these difficulties remained evident even after accounting for anxiety, depression, and age. Furthermore, sleep disturbance was more strongly linked to anxiety in autistic individuals, pointing to a potentially unique interplay between sleep and mental health in this population. Older autistic adults may be at elevated risk, emphasising the need for lifespan‐oriented and autism‐informed clinical interventions. These findings highlight the importance of integrating sleep assessments into routine mental health care for autistic adults, particularly as they age. Given the bidirectional relationship between sleep problems and psychiatric symptoms, targeting sleep may offer a valuable pathway to improving overall well‐being in this population. These findings position sleep as a potentially modifiable target within broader interventions aimed at improving mental health and quality of life in ageing autistic populations. Future research using longitudinal designs, objective sleep measures, and more diverse, representative samples will be essential to advance evidence‐based, personalised care.

## Author Contributions

Authors F.H., R.A.C., and G.R.S. conceived the AgeWellAutism study. G.R.S. designed the online survey and selected materials. G.R.S. conceived the current study. S.R., A.B., and G.R.S. conducted analyses. S.R. wrote the manuscript under the supervision of G.R.S. A.B., F.H., and R.A.C. reviewed the final draft. All authors have read and approved the final manuscript.

## Conflicts of Interest

The authors declare no conflicts of interest.

## Supporting information


Supporting Information S1

## Data Availability

The data that support the findings of this study are available on request from the corresponding author. The data are not publicly available due to privacy or ethical restrictions.
