# Supplementary material for: Sleep Problems and Mental Health in Middle‐Aged and Older Autistic and Non‐Autistic Adults
Source: Int J Geriatr Psychiatry. 2026 Oct 7;41(10):e70259. doi: 10.1002/gps.70259 (PMC13645436; doi:10.1002/gps.70259)
Supplement: Supplementary file 1 [file GPS-41-e70259-s001.docx]

| Supplementary Table 1. Gender differences in frequencies of sleep problem domains, split by autistic and non-autistic groups. | | | | | | | | | | | | |
| --- | --- | --- | --- | --- | --- | --- | --- | --- | --- | --- | --- | --- |
|  |  |  | **Sleep Problems Score** | | | | | | | | **Group Difference** | **Effect Size** |
|  |  |  | **No difficulty** | | **Some difficulty** | | **Moderate difficulty** | | **Severe Difficulty** | |  |  |
| **Sleep Quality** | *Autistic* | *Men* | 64 | (51.6%) | 49 | (39.5%) | 11 | (8.9%) | 0 | - | χ2=5.70, *p=*.058 | v=.148 |
|  |  | *Women* | 73 | (53.3%) | 40 | (29.2%) | 24 | (17.5%) | 0 | - |  |  |
|  | *non-Autistic* | *Men* | 78 | (92.9%) | 5 | (6.0%) | 1 | (1.2%) | 0 | - | χ2=.386, *p=*.824 | v=.048 |
|  |  | *Women* | 75 | (90.4%) | 7 | (8.4%) | 1 | (1.2%) | 0 | - |  |  |
| **Sleep Latency** | *Autistic* | *Men* | 8 | (6.5%) | 18 | (14.6%) | 70 | (56.9%) | 27 | (22.0%) | χ2=1.51, *p=*.680 | v=.160 |
|  |  | *Women* | 5 | (3.6%) | 13 | (9.5%) | 71 | (51.8%) | 48 | (35.0%) |  |  |
|  | *non-Autistic* | *Men* | 2 | (2.4%) | 25 | (29.8%) | 41 | (48.8%) | 16 | (19.0%) | χ2=6.65, *p=*.084 | v=.095 |
|  |  | *Women* | 4 | (4.8%) | 27 | (32.5%) | 34 | (41.0%) | 18 | (21.7%) |  |  |
| **Sleep Duration** | *Autistic* | *Men* | 69 | (56.1%) ╪ | 34 | (27.6%) | 17 | (13.8%) ╪ | 3 | (2.4%) ╪ | χ2=13.39, *p=*.004** | v=.227 |
|  |  | *Women* | 49 | (35.8%) ╪ | 44 | (32.1%) | 34 | (24.8%) ╪ | 10 | (7.3%) ╪ |  |  |
|  | *non-Autistic* | *Men* | 48 | (57.1%) | 25 | (29.8%) | 11 | (13.1%) | 0 | - | χ2=.204, *p=*.903 | v=.035 |
|  |  | *Women* | 49 | (59.0%) | 25 | (30.1%) | 9 | (10.8%) | 0 | - |  |  |
| **Sleep Efficiency** | *Autistic* | *Men* | 73 | (59.3%) | 25 | (20.3%) | 25 | (20.3%) | 0 | - | χ2=3.05, *p=*.217 | v=.108 |
|  |  | *Women* | 67 | (48.9%) | 38 | (27.7%) | 32 | (23.4%) | 0 | - |  |  |
|  | *non-Autistic* | *Men* | 45 | (53.6%) | 32 | (38.1%) | 7 | (8.3%) | 0 | - | χ2=.387, *p=*.824 | v=.048 |
|  |  | *Women* | 47 | (56.6%) | 31 | (37.3%) | 5 | (6.0%) | 0 | - |  |  |
| **Sleep Disturbance** | *Autistic* | *Men* | 3 | (2.4%) | 68 | (54.8%) ╪ | 50 | (40.3%) ╪ | 3 | (2.4%) ╪ | χ2=11.39, *p=*.010** | v=.209 |
|  |  | *Women* | 1 | (0.7%) | 52 | (38.0%) ╪ | 73 | (53.3%) ╪ | 11 | (8.0%) ╪ |  |  |
|  | *non-Autistic* | *Men* | 16 | (19.0%) | 60 | (71.4%) | 8 | (9.5%) | 0 | - | χ2=.761, *p=*.683 | v=.068 |
|  |  | *Women* | 12 | (14.5%) | 64 | (77.1%) | 7 | (8.4%) | 0 | - |  |  |
| **Medication Use** | *Autistic* | *Men* | 73 | (58.9%) | 43 | (34.7%) | 8 | (6.5%) | 0 | - | χ2=2.19, *p=*.335 | v=.092 |
|  |  | *Women* | 78 | (56.9%) | 43 | (31.4%) | 16 | (11.7%) | 0 | - |  |  |
|  | *non-Autistic* | *Men* | 64 | (76.2%) | 18 | (21.4%) | 2 | (2.4%) | 0 | - | χ2=3.07, *p=*.216 | v=.136 |
|  |  | *Women* | 70 | (84.3%) | 13 | (15.7%) | 0 | - | 0 | - |  |  |
| **Daytime Dysfunction** | *Autistic* | *Men* | 6 | (4.8%) | 68 | (54.8%) ╪ | 42 | (33.9%) ╪ | 8 | (6.5%) | χ2=13.38, *p=*.004** | v=.226 |
|  |  | *Women* | 3 | (2.2%) | 48 | (35.0%) ╪ | 73 | (53.3%) ╪ | 13 | (9.5%) |  |  |
|  | *non-Autistic* | *Men* | 22 | (26.2%) | 46 | (54.8%) | 14 | (16.7%) | 2 | (2.4%) | χ2=4.63, *p=*.201 | v=.116 |
|  |  | *Women* | 32 | (38.6%) | 40 | (48.2%) | 11 | (13.3%) | 0 | - |  |  |
| Note: ╪ indicates significant adjusted residual value. FDR adjusted a-value = * *p<*.029, ** *p<*.01, *** *p<*.001. | | | | | | | | | | | | |

| *Supplementary Table 2. Descriptive statistics, group differences, and interactions gender and autism group membership on sleep problems.* | | | | | | | | | | | | |
| --- | --- | --- | --- | --- | --- | --- | --- | --- | --- | --- | --- | --- |
|  |  | **Autistic Women (n=137)** | | **Autistic Men (n=123)** | | **Non-Autistic Women (n=84)** | | **Non-Autistic Men (n=83)** | | **Autism Main Effect** | **Gender Main Effect** | **Group x Gender Interaction** |
| **Sleep Quality Index** (max score = 21) | *M (SD)* | 8.54 | (3.06) | 7.07 | (2.68) | 4.76 | (1.99) | 5.15 | (2.24) | F(1,423)=121.29, *p<*.001*** | F(1,423)=4.26, *p=*.040 | F(1,423)=12.89, *p<*.001*** |
|  | *[95% CI]* | [8.02-9.05] | | [6.59-7.55] | | [4.32-5.19] | | [4.66-5.64] | |  |  |  |
|  | *Mix-Max* | 1 -16 | | 1 - 14 | | 1 - 11 | | 1 - 14 | |  |  |  |
| **Depression** (max score = 27, cut-off ≥10) | *M (SD)* | 10.05 | (5.50) | 9.57 | (5.23) | 3.13 | (2.51) | 2.86 | (2.29) | F(1,423)=237.35, *p<*.001 | F(1,423)=0.73, *p=*.393 | F(1,423)=0.05, *p=*.816 |
|  | *[95% CI]* | [9.12-10.98] | | [8.64-10.50] | | [2.59-3.68] | | [2.36-3.35] | |  |  |  |
|  | *Mix-Max* | 0 - 18 | | 0 - 18 | | 0 - 11 | | 1 - 13 | |  |  |  |
| **Anxiety** (max score = 21, cut-off ≥10) | *M (SD)* | 6.36 | (3.34) | 6.33 | (3.36) | 2.57 | (1.65) | 2.40 | (1.56) | F(1,423)=192.98, *p<*.001 | F(1,423)=0.15, *p=*.695 | F(1,423)=0.03, *p=*.851 |
|  | *[95% CI]* | [5.79-6.92] | | [5.73-6.93] | | [2.21-2.93] | | [2.07-2.74] | |  |  |  |
|  | *Mix-Max* | 1 -15 | | 1 - 18 | | 0 - 11 | | 0 - 11 | |  |  |  |
| **PTSD** (max score = 24, cut-off ≥17) | *M (SD)* | 14.42 | (6.04) | 9.56 | (6.04) | 4.22 | (4.44) | 2.51 | (2.41) | F(1,423)=267.31, *p<*.001*** | F(1,423)=42.90, *p<*.001*** | F(1,423)=9.97, *p=*.002** |
|  | *[95% CI]* | [13.48-15.37] | | [8.49-10.64] | | [3.25-5.19] | | [1.99-3.03] | |  |  |  |
|  | *Mix-Max* | 0 - 24 | | 0 - 24 | | 0 - 23 | | 0 - 13 | |  |  |  |
| Note: FDR adjusted a-value = * *p<*.029, ** *p<*.01, *** *p<*.001. | | | | | | | | | | | | |

| *Supplementary Table 3. Co-variate adjusted descriptive statistics and group differences of sleep problems and mental health problems of the autistic and non-autistic groups.* | | | | | | | |
| --- | --- | --- | --- | --- | --- | --- | --- |
|  |  | **Autistic group (n=265)** | | **non-Autistic group (n=167)** | | **Group Difference** | **Effect Size** |
| **Sleep Quality Index** (max score = 21) | *Adjusted M (SE)* | 7.21 | (0.16) | 5.98 | (0.22) | F(1,425)=16.20, *p<*.001*** | η^2^=0.37 |
|  | *[95% CI]* | [6.89-7.52] | | [5.55-6.41] | |  |  |
|  | *Min-Max* | 1 - 16 | | 1 - 14 | |  |  |
| Note: Covariates include depression symptoms, anxiety symptoms, PTSD symptoms and age. FDR adjusted a-value = * *p<*.029, ** *p<*.01, *** *p<*.001. | | | | | | | |

| *Supplementary Table 4. Co-variate adjusted descriptive statistics, group differences, and interactions gender and autism group membership on sleep problems.* | | | | | | | | | | | | |
| --- | --- | --- | --- | --- | --- | --- | --- | --- | --- | --- | --- | --- |
|  |  | **Autistic Women (n=137)** | | **Autistic Men (n=123)** | | **Non-Autistic Women (n=84)** | | **Non-Autistic Men (n=83)** | | **Autism Main Effect** | **Gender Main Effect** | **Group x Gender Interaction** |
| **Sleep Quality Index** (max score = 21) | *Adjusted M (SE)* | 7.80 | (0.23) | 6.59 | (0.22) | 5.71 | (0.27) | 6.12 | (0.28) | F(1,419)=17.97, *p<*.001*** | F(1,419)=2.70, *p=*.101 | F(1,419)=12.28, *p<*.001*** |
|  | *[95% CI]* | [7.34-8.26] | | [6.16-7.02] | | [5.17-6.24] | | [5.55-6.81] | |  |  |  |
|  | *Mix-Max* | 1 -16 | | 1 - 14 | | 1 - 11 | | 1 - 14 | |  |  |  |
| Note: Covariates include depression symptoms, anxiety symptoms, PTSD symptoms and age. FDR adjusted a-value = * *p<*.029, ** *p<*.01, *** *p<*.001. | | | | | | | | | | | | |

| *Supplementary Table 5. Age group differences in frequencies of sleep problem domains, split by autistic and non-autistic groups.* | | | | | | | | | | | | | |
| --- | --- | --- | --- | --- | --- | --- | --- | --- | --- | --- | --- | --- | --- |
|  |  | **Sleep Problems Score** | | | | | | | | | | **Group Difference** | **Effect Size** |
|  |  |  | **No difficulty** | | **Some difficulty** | | **Moderate difficulty** | | **Severe Difficulty** | | |  |  |
| **Sleep Quality** | *Autistic* | *Midlife* | 95 | (59.7%) ╪ | 49 | (30.8%) | 15 | (9.4%) ╪ | 0 | - | χ2=9.55, *p=*.008** | | v=.190 |
|  |  | *Old Age* | 45 | (42.5%) ╪ | 40 | (37.7%) | 19 | (19.8%) ╪ | 0 | - |  |  |  |
|  | *non-Autistic* | *Midlife* | 98 | (92.5%) | 7 | (6.6%) | 1 | (0.9%) | 0 | - | χ2=0.31, *p=*.854 | | v=.043 |
|  |  | *Old Age* | 55 | (90.2%) | 5 | (8.2%) | 1 | (1.6%) | 0 | - |  |  |  |
| **Sleep Latency** | *Autistic* | *Midlife* | 12 | (7.6%) | 22 | (13.9%) | 82 | (51.9%) | 42 | (26.6%) | χ2=6.02, *p=*.111 | | v=.151 |
|  |  | *Old Age* | 2 | (1.9%) | 10 | (9.4%) | 59 | (55.7%) | 35 | (33.0%) |  |  |  |
|  | *non-Autistic* | *Midlife* | 6 | (5.7%) | 36 | (34.0%) | 45 | (42.5%) | 19 | (17.9%) | χ2=5.43, *p=*.143 | | v=.180 |
|  |  | *Old Age* | 0 | - | 16 | (26.2%) | 30 | (49.2%) | 15 | (24.6%) |  |  |  |
| **Sleep Duration** | *Autistic* | *Midlife* | 81 | (51.3%) ╪ | 38 | (24.1%) ╪ | 33 | (20.9%) | 6 | (3.8%) | χ2=9.02, *p=*.029* | | v=.185 |
|  |  | *Old Age* | 38 | (35.8%) ╪ | 41 | (38.7%) ╪ | 20 | (18.9%) | 7 | (6.6%) |  |  |  |
|  | *non-Autistic* | *Midlife* | 55 | (51.9%) | 36 | (34.0%) | 15 | (14.2%) | 0 | - | χ2=4.63, *p=*.099 | | v=.167 |
|  |  | *Old Age* | 42 | (68.9%) | 14 | (23.0%) | 5 | (8.2%) | 0 | - |  |  |  |
| **Sleep Efficiency** | *Autistic* | *Midlife* | 93 | (58.9%) ╪ | 28 | (17.7%) ╪ | 37 | (23.4%) | 0 | - | χ2=8.31, *p=*.016* | | v=.177 |
|  |  | *Old Age* | 49 | (46.2%) ╪ | 35 | (33.0%) ╪ | 22 | (20.8%) | 0 | - |  |  |  |
|  | *non-Autistic* | *Midlife* | 50 | (47.2%) ╪ | 48 | (45.3%) ╪ | 8 | (7.5%) | 0 | - | χ2=7.75, *p=*.021* | | v=.215 |
|  |  | *Old Age* | 49 | (46.2%) ╪ | 35 | (33.0%) ╪ | 22 | (20.8%) | 0 | - |  |  |  |
| **Sleep Disturbance** | *Autistic* | *Midlife* | 4 | (2.5%) | 94 | (59.1%) ╪ | 61 | (38.4%) ╪ | 0 | - ╪ | χ2=44.97, *p<*.001*** | | v=.412 |
|  |  | *Old Age* | 0 | - | 28 | (26.4%) ╪ | 64 | (60.4%) ╪ | 14 | (13.2%) ╪ |  |  |  |
|  | *non-Autistic* | *Midlife* | 27 | (25.5%) ╪ | 72 | (67.9%) ╪ | 7 | (6.6%) | 0 | - | χ2=16.51, *p<*.001*** | | v=.314 |
|  |  | *Old Age* | 1 | (1.6%) ╪ | 52 | (85.2%) ╪ | 8 | (13.1%) | 0 | - |  |  |  |
| **Medication Use** | *Autistic* | *Midlife* | 102 | (64.2%) ╪ | 46 | (28.9%) ╪ | 11 | (6.9%) | 0 | - | χ2=8.05, *p=*.018* | | v=.174 |
|  |  | *Old Age* | 50 | (47.2%) ╪ | 42 | (39.6%) ╪ | 14 | (13.2%) | 0 | - |  |  |  |
|  | *non-Autistic* | *Midlife* | 88 | (83.0%) | 18 | (17.0%) | 0 | - | 0 | - | χ2=4.15, *p=*.126 | | v=.158 |
|  |  | *Old Age* | 46 | (75.4%) | 13 | (21.3%) | 2 | (3.3%) | 0 | - |  |  |  |
| **Daytime Dysfunction** | *Autistic* | *Midlife* | 4 | (2.5%) | 89 | (56.0%) ╪ | 54 | (34.0%) ╪ | 12 | (7.5%) | χ2=23.53, *p<*.001*** | | v=.298 |
|  |  | *Old Age* | 5 | (4.7%) | 28 | (26.4%) ╪ | 64 | (60.4%) ╪ | 9 | (8.5%) |  |  |  |
|  | *non-Autistic* | *Midlife* | 41 | (38.7%) ╪ | 53 | (50.0%) | 12 | (11.3%) ╪ | 0 | - ╪ | χ2=9.79, *p=*.020* | | v=.242 |
|  |  | *Old Age* | 13 | (21.3%) ╪ | 33 | (54.1%) | 13 | (21.3%) ╪ | 2 | (3.3%) ╪ |  |  |  |
| Note: ╪ indicates significant adjusted residual value. FDR adjusted a-value = * *p<*.029, ** *p<*.01, *** *p<*.001. | | | | | | | | | | | | | |

| *Supplementary Table 6. Descriptive statistics, group differences, and interactions of age and autism group membership on sleep problems.* | | | | | | | | | | | | |
| --- | --- | --- | --- | --- | --- | --- | --- | --- | --- | --- | --- | --- |
|  |  | **Autistic Midlife (n=158)** | | **Autistic Old Age (n=107)** | | **non-Autistic Midlife (n=106)** | | **non-Autistic Old Age (n=61)** | | **Autism Main Effect** | **Age Main Effect** | **Group x Age Interaction** |
| **Sleep Quality Index** (max score = 21) | *M (SD)* | 7.13 | (2.65) | 8.93 | (3.06) | 4.74 | (2.10) | 5.32 | (2.12) | F(1,427)=54.23, *p<*.001*** | F(1,427)=20.51, *p<*.001*** | F(1,427)=5.33, *p=*.021* |
|  | *[95% CI]* | [6.72-7.55] | | [8.34-9.52] | | [4.34-5.15] | | [4.78-5.87] | |  |  |  |
|  | *Mix-Max* | 1 -14 | | 1 - 16 | | 1 - 11 | | 1 - 14 | |  |  |  |
| Note: FDR adjusted a-value = * *p<*.029, ** *p<*.01, *** *p<*.001. | | | | | | | | | | | | |

| *Supplementary Table 7. Co-variate adjusted descriptive statistics, group differences, and interactions of age and autism group membership on sleep problems.* | | | | | | | | | | | | |
| --- | --- | --- | --- | --- | --- | --- | --- | --- | --- | --- | --- | --- |
|  |  | **Autistic Midlife (n=158)** | | **Autistic Old Age (n=107)** | | **non-Autistic Midlife (n=106)** | | **non-Autistic Old Age (n=61)** | | **Autism Main Effect** | **Age Main Effect** | **Group x Age Interaction** |
| **Sleep Quality Index** (max score = 21) | *M (SD)* | 7.30 | (0.24) | 7.10 | (0.34) | 6.28 | (0.28) | 5.39 | (0.40) | F(1,423)=18.96, *p<*.001*** | F(1,423)=1.59, *p*=.208 | F(1,423)=1.83, *p=*.236 |
|  | *[95% CI]* | [6.83-7.77] | | [6.43-7.78] | | [5.72-6.85] | | [4.61-6.18] | |  |  |  |
|  | *Mix-Max* | 1 -14 | | 1 - 16 | | 1 - 11 | | 1 - 14 | |  |  |  |
| Note: Covariates include depression symptoms, anxiety symptoms, PTSD symptoms and age. FDR adjusted a-value = * *p<*.029, ** *p<*.01, *** *p<*.001. | | | | | | | | | | | | |
